# HIV-Derived ssRNA Binds to TLR8 to Induce Inflammation-Driven Macrophage Foam Cell Formation

**DOI:** 10.1371/journal.pone.0104039

**Published:** 2014-08-04

**Authors:** Mark A. Bernard, Xinbing Han, Sonya Inderbitzin, Ifunanya Agbim, Hui Zhao, Henry Koziel, Souvenir D. Tachado

**Affiliations:** 1 Division of Pulmonary, Critical Care, and Sleep Medicine; Department of Medicine, Beth Israel Deaconess Medical Center and Harvard Medical School, Boston, Massachusetts, United States of America; 2 From Department of Respiratory Medicine, The Second Hospital of Shanxi Medical University, Taiyuan, PR China; Brigham and Women's Hospital, Harvard Medical School, United States of America

## Abstract

Even though combined anti-retroviral therapy (cART) dramatically improves patient survival, they remain at a higher risk of being afflicted with non-infectious complications such as cardiovascular disease (CVD). This increased risk is linked to persistent inflammation and chronic immune activation. In this study, we assessed whether this complication is related to HIV-derived ssRNAs inducing in macrophages increases in TNFα release through TLR8 activation leading to foam cell formation. HIV ssRNAs induced foam cell formation in monocyte-derived macrophages (MDMs) in a dose-dependent manner. This response was reduced when either endocytosis or endosomal acidification was inhibited by dynasore or chloroquine, respectively. Using a flow cytometry FRET assay, we demonstrated that ssRNAs bind to TLR8 in HEK cells. In MDMs, ssRNAs triggered a TLR8-mediated inflammatory response that ultimately lead to foam cell formation. Targeted silencing of the *TLR8* and *MYD88* genes reduced foam cell formation. Furthermore, foam cell formation induced by these ssRNAs was blocked by an anti-TNFα neutralizing antibody. Taken together in MDMs, HIV ssRNAs are internalized; bind TLR8 in the endosome followed by endosomal acidification. TLR8 signaling then triggers TNFα release and ultimately leads to foam cell formation. As this response was inhibited by a blocking anti-TNFα antibody, drug targeting HIV ssRNA-driven TLR8 activation may serve as a potential therapeutic target to reduce chronic immune activation and inflammation leading to CVD in HIV+ patients.

## Introduction

Increased risk of atherosclerosis and coronary heart disease (CHD) is a well-recognized clinical problem in HIV-infected patients [1], [2]. HIV survivors in the United States aged 50 and older have increased significantly with reduced AIDS-related morbidity and mortality due to the introduction of combination anti-retroviral therapy (cART) [3] however, these anti-retroviral drugs failed to fully restore health in HIV-infected individuals. As this population continues to age, CHD becomes increasingly an important issue. This issue is closely associated with inflammation that persists in cART-treated HIV+ individuals despite undetectable plasma viremia levels. Careful assessment of heightened CHD risk in HIV+ patients is required to fully understand the underlying causes. CHD incidence in HIV afflicted individuals is three-fold greater than that in the general population [2], [4]. However, it is not yet clear whether cardiovascular complications are a consequence of HIV infection itself or due to long-term use of HAART, or a combination of both. Interestingly, clinical presentations of CHD in HIV infection are distinct from CHD due to traditional risk factors. HIV patients are a decade younger with a mean of 50 years, and unlike non-HIV patients tend to have a single vessel affected rather than multiple vessels [5]. Moreover, in HIV patients whose infection is controlled without receiving cART (“elite controllers”), they also have more extensive carotid narrowing than age-matched controls [6]. This association argues for a direct effect by HIV-associated factors in inducing cardiovascular disease [6]. HIV infection by itself is implicated to associate with an increased risk of myocardial infarction based on the results of the Strategies for Management of Anti-Retroviral Therapy (SMART) study. Namely, patients undergoing episodic antiretroviral therapy had an increased risk of cardiovascular events than those undergoing continuous therapy [7]. Taken together, these data show that HIV infection by itself markedly contributes to atherosclerotic cardiovascular disease independent of other traditional risk factors and cART.

The underlying mechanisms of early atherosclerosis in HIV disease are not well understood, but similarly may be closely linked to increased vascular inflammation. Toll-like receptors (TLRs) are a superfamily of pathogen and viral constituent pattern recognition receptors (PRRs) that could play a central role in pathogen-induced atherosclerosis [8]. TLRs 1, 2, 4, 5 and 6 are highly upregulated in human atheroma compared to healthy controls. Associated with this change, activated NF-κB co-localizes in cells within atheromatous plaques expressing TLR2 or TLR4 [9]. In addition, functional studies confirmed that excised and cultured human carotid plaques secrete TNFα and IFNγ in response to treatment with the TLR4 and TLR9 ligands: LPS and CpG DNA, respectively [10]. Foam cell formation is implicated to be dependent on TLR2 activation as suggested in a TLR2-deficient mouse model infected with *Chlamydia pneumonia* [11]. In addition, treatment of macrophages with either LPS (TLR4 agonist), CpG DNA (TLR9 agonist) promoted foam cell formation [11]–[14]. These findings suggest that recognition of bacterial pathogen associated molecular patterns (PAMPs) by TLRs is involved in eliciting bacteria-induced foam cell formation.

Monocytes are precursors of macrophages within atherosclerotic regions, that can transdifferentiate into lipid-laden foam cells [15]–[17]. Macrophages play a critical role in the pathogenesis of atherosclerosis. They are also a target of invading pathogens including HIV that interfere with their immune function [18]. Plasma levels of a number of proinflammatory cytokines are increased in HIV-infected persons [19], and their elevated levels persist during HIV infection due to low-level viral replication, even in patients receiving cART [20], [21]. In addition, macrophages associated with atherosclerotic lesions are a major source of both proinflammatory cytokines including TNFα as well as chemokines that could direct monocyte migration into these vascular lesions. The inflammatory cascade activated by HIV has clear and diffuse circumferential effects along the vascular endothelium, which are distinct from accelerated atherosclerosis [22], [23]. Therefore, changes that occur in monocytes and macrophages during HIV infection are likely to impact on the atherogenic process. HIV interferes with the ability of macrophages to handle excessive cholesterol by inhibiting cholesterol efflux, which could affect initiation and progression of atherosclerotic events.

Using HEK-293 cells overexpressing TLR8 and clinically relevant human healthy monocyte-derived macrophages (MDMs), as well as primary alveolar macrophages (AMs), we show in this report that HIV infection per se is sufficient to induce foam cell formation. HIV-derived ssRNA binding to TLR8 induces foam cell formation in part through inflammatory TNFα release. TNFα involvement is likely since blocking TNFα down-regulates foam cell formation. Foam cell induction by ssRNA is dependent on dynamin-mediated endosomal uptake as well as intravesicular acidification. Furthermore, ssRNA TLR8 binding and internalization leads to TNFα release and foam cell formation since TLR8 gene silencing reduced this response. These results suggest that proinflammatory factors including TNFα induced by HIV derived ssRNA may activate monocytes to potentiate early atherogenic processes in HIV+ individuals.

## Materials and Methods

### Reagents

ssRNA40/LyoVec and ssRNA41/Lyovec were purchased from InvivoGen (San Diego, CA). Lipid A (the biologically active component of LPS, and specific TLR4 ligand) from *Escherichia coli* F583 Rd mutant, protease inhibitor cocktail and phorbol myristic acid (PMA) were purchased from Sigma Chemical Company (St Louis, MO). Bacterial lipopeptide (BLP) was from EMD Millipore (Billerica, MA). Oxidized LDL was from Biomedical Technologies, Inc. (Ward Hill, MA). Dynasore, and chloroquine were purchased from Calbiochem (San Diego, CA). Paraformaldehyde solution (4% in Phosphate Buffered Saline) was from Fisher Scientific. BODIPY 493/503 dye was from Life Technologies (Carlsbad, CA). Saponin (molecular biology grade) was from Sigma. Phosphate Buffered Saline without calcium or magnesium was from Cellgro (Herndon, VA). Nef, was purchased from Trinity Biotech (Wicklow, Ireland). The phosphorothioate oligoribonucleotides, GU-rich ssRNA40 from LTR of HIV (5′-GCCCGUCUGUUGUGUGACUC-3′, and GA-rich ssRNA41 (5′-GCCCGACAGAAGAGAGACAC-3′), were purchased from Invivogen, San Diego, CA. Phosphorothioate oligoribonucleotide conjugate Alexa 546-ssRNA40 (5′-Alexa Fluor 546 GCCCGUCUGUUGUGUGACUC-3′) was from Integrated DNA Technologies (Coralville, IA). The phosphorothioate oligoribonucleotides antagomir99 (5′-mCmAmAmCmAmGmAmCmGmGmGmCmAmCmAmCmAmCmUmAmC-3′), HIV vmiR99 (5′-PO_4_-GUAGUGUGUGCCCGUCUGUmUmG-3′) and HIV vmiR-TAR (5′-PO_4_-CUAACUAGGGAACCCACUmGmC-3′) were from Integrated DNA Technologies (Coralville, IA); “m” denotes 2′-O-methylation. Oligoribonucleotides were prepared (50 µg/mL) in LyoVec according to the manufacturer’s instructions (Invivogen) before each experiment.

### Antibodies

Anti-TLR8-PE antibodies were purchased from Imgenex, (San Diego, CA). Anti-MyD88, and β-actin antibodies were purchased from Cell Signaling (Beverly, MA). Fluorescent conjugates of monoclonal antibody against TLR8/CD288 (clone 44C143; mouse IgG1/κ), Alexa Fluor 488 conjugate of mouse IgG1/κ isotype control (clone MOPC-31C) were from Imgenex (San Diego, CA). Cytokine ELISA kits were from R&D Systems (Minneapolis, MN). HIV-1 p24 antigen ELISA kit was from Zeptometrix (Franklin, MA).

### Alveolar macrophages (AM)

To determine the clinical relevance of the study, select experiments were carried out using human AM. Recruited healthy subjects had no active pulmonary disease and normal spirometry. They were confirmed to be HIV seronegative by ELISA and had no known risk factors for HIV infection. Using standard techniques, bronchoalveolar lavage (BAL) was performed to obtain lung immune cells [24]. All procedures were performed with written informed consent on adults following protocols approved by Beth Israel Deaconess Medical Center Institutional Review board and Committee for Clinical Investigations. Cells were separated from the pooled BAL fluid and AM were isolated [18]. AM were isolated by adherence to culture plates in medium (RPMI 1640 containing 10% heat-inactivated fetal bovine serum, 2 mM glutamine, 100 U/mL penicillin, 100 µg/mL streptomycin and 250 ng/mL amphotericin B), and yielded cells that were >98% viable as determined by trypan blue dye exclusion, and demonstrated >95% positive nonspecific esterase staining.

### Monocyte-derived macrophages (MDMs)

Healthy individuals were confirmed to be HIV seronegative by ELISA and had no known risk factors for HIV infection. Using standard techniques, venipuncture was performed to obtain peripheral blood. All procedures were performed with written informed consent on adults following protocols approved by Beth Israel Deaconess Medical Center institutional review board and Committee for Clinical Investigations. Healthy MDM was isolated from buffy coat of healthy subjects using Percol Hypaque and cultured for 7–10 days in medium (RPMI 1640 containing 10% heat-inactivated fetal bovine serum, 2 mM glutamine, 100 U/mL penicillin, 100 µg/mL streptomycin and 250 ng/mL amphotericin B) containing macrophage colony stimulating factor (M-CSF), and non-adherent cells were washed away followed by addition of fresh medium prior to experimentation.

### Cells and Macrophage cell lines

HEK-293 stable cell line expressing full-length human TLR8 was from Imgenex. Macrophages were differentiated from human promonocytic THP-1 (American Tissue and Cell Company, ATCC). THP-1 cells were harvested during exponential growth phase, pelleted, resuspended and then incubated in complete medium (RPMI 1640 containing 10% heat-inactivated fetal calf serum (FCS), 2 mM glutamine, 100 U/ml penicillin and 100 µg/ml streptomycin). To induce macrophage differentiation, THP-1 cells were incubated with 100 nM PMA at 37°C in a humidified atmosphere containing 5% CO_2_ for 24 h. Adherent cells were then washed three times with medium (to remove PMA) and incubated (37°C, 5% CO_2_) in complete medium (without PMA) for use in experiments.

### Western blot analysis

Western blotting was performed as described [25]. Briefly, adherent human macrophages were treated with indicated reagents, washed 2x with ice-cold PBS (pH 7.4). Cells were lysed in lysis buffer containing 25 mM Tris-HCl (pH 7.6), 150 mM NaCl, 1% NP-40, 1% sodium deoxycholate, 0.1% SDS and protease inhibitor cocktail (Sigma Chemicals; St. Louis, MO), placed on ice for 20 min. Cells were harvested by scraping, followed by centrifugation at 4°C for 15 min at 14,000 rpm. Equal amounts of cell lysates were subjected to SDS/PAGE and Western blot analysis with designated antibodies and detected by enhanced chemiluminescence (ECL) detection system (Amersham Biosciences; Piscataway, NJ). Resolved bands were quantified by densitometry (Amersham Biosciences; Piscataway, NJ).

### Small Interfering RNA (siRNA)- mediated knockdown in macrophages

To determine the functional relevance of TLR8 signaling pathway in foam cell formation, RNAi-mediated knockdown of TLR8 was performed using synthetic duplex RNA oligonucleotides including On-Target Plus Smart Pool short interfering RNA (siRNA) TLR8 (Thermo Scientific). Target sequences for TLR8 were GAACGGAAAUCCCGGUAUA, CAGAAUAGCAGGCGUAACA, GUGCAGCAAUCGUCGACUA, and CUUCCAAACUUAUCGACUA. The non-targeting irrelevant siRNA (ON-TARGET plus Non-targeting siRNA#1 from Thermo Scientific) was used as control. Macrophages were electroporated with 100 nM siRNA using Amaxa Nucleofector system following the manufacturer’s protocol (Amaxa). TLR8-mediated knockdown was determined by Western blot probed with anti-TLR8, or flow cytometry using anti-TLR8-PE 24–48 h after transfection.

### Binding assay by flow cytometry – fluorescence resonance energy transfer (FC-FRET)

HEK-293 cells expressing TLR8 were plated in 6-well plates and grown in DMEM containing glucose (4.5 g/L), 10% FBS, L-glutamine (4 mM), sodium pyruvate (1 mM), penicillin (100 units/mL), streptomycin (100 µg/mL) and blasticidin (10 µg/mL) in humidified atmosphere (37°C, 5% CO_2_). After aspiration, fresh medium was applied (1.0 mL/well) without blasticidin. Oligonucleotide ssRNA40-Alexa 546 was prepared at 50 µg/mL in LyoVec (Invivogen, San Diego, CA) according to manufacturer’s instructions and applied to wells (1.0 µg/mL) for 20 min at 37°C, 5% CO_2_. Medium was aspirated, and cells were treated with trypsin/EDTA. Medium (2 mL) was applied to the cell suspension and pelleted in centrifuge tubes. Cell pellets were re-suspended in paraformaldehyde (4% in PBS) for 20 min at RT and then washed once in PBS. Cell pellets were re-suspended and permeabilized in PBS containing 0.1% saponin for 15 min at RT. Cells were pelleted and supernatant aspirated. Cells were re-suspended in PBS/0.1% saponin containing anti-TLR8 Alexa 488 or mouse IgG1/κ Alexa 488 (40 µg/mL) in the dark for 30 min at RT. Cells were washed in PBS/0.1% saponin, centrifuged, re-suspended in PBS containing 0.5% paraformaldehyde and placed in the dark at 4°C. Cells were analyzed using a SORP LSR II flow cytometer system (BD Biosciences, San Jose, CA) configured with a solid state 50-mW 488-nm laser and a Coherent Compass 25-mW 561-nm laser. For direct detection of labeled cells, Alexa 488 was excited (488-nm laser) through the 505-nm longpass dichroic mirror and emission detected through the 530/30 bandpass filter. Direct detection of Alexa 546 was achieved by exciting with the 561-nm laser with emission detected through the 585/42 bandpass filter. For FRET assay, the 561-nm laser was disabled and its light path physically blocked to ensure that FRET excitation was provided exclusively by the 488-nm laser. FRET Donor (Alexa 488) was detected using the 488-nm laser with emission through the 505-nm longpass dichroic mirror and emission detected through the 530/30 bandpass filter. The FRET Acceptor (Alexa 546) was detected via 550-nm longpass dichroic mirror and emission through the 575/26 bandpass filter. Thus the presence of antibody-Alexa 488 and ssRNA-Alexa 546 was confirmed by direct excitation using 488-nm and 561-nm lasers, respectively. After disabling the 561-nm laser, FRET was assessed by exciting with the 488-nm laser and detecting emission by FRET Donor (Alexa 488) and FRET Acceptor (Alexa 546). A minimum of 10,000 events was collected for each sample, and data were analyzed using FCS Express Flow Cytometry software (De Novo Software, Los Angeles, CA).

### TLR analysis by flow cytometry

Cell surface expression of TLRs was determined by Epics XL flow cytometer (Beckman/Coulter, Miami, FL) with laser power of 5.76 mW. The instrument was calibrated before each measurement with standardized fluorescent particles (Immunocheck; AMAC, Inc. Westbrook, ME). Fluorescence signals of the cells were measured simultaneously by 3 photomultiplier tubes and optical filters and shown as the mean of the log fluorescence intensity of the cell population within each gate. Macrophages were incubated with an anti-TLR4 antibody on ice for 60 min, washed three times, incubated with a FITC-conjugated secondary antibody for 30 min on ice protected from light, fixed in Optilyse (Beckman/Coulter, Miami, FL) at RT for 5–10 min, and analyzed by flow cytometry. Human macrophages were first identified by the characteristic forward and side scatter parameters on unstained cells, and confirmed by staining with PE-conjugated primary anti-human HLA-DR (Beckman/Coulter, Miami, FL). Data were expressed as a mean relative fluorescence units (RFU) and the percentage of cells staining positive. Isotype primary conjugated antibodies served as a negative control. Samples were prepared and analyzed in duplicate, and a minimum of 5,000 cells was counted for each sample.

### ELISA

After cell stimulation, supernatants were collected, centrifuged to remove cellular debris, and assayed immediately or stored at –80°C until assayed. Cytokine measurements were performed using commercially available ELISA (R&D Systems, Minneapolis, MN) following manufacturer’s instructions, and absorbance (450 nm) was measured using an Emax ELISA plate reader with multi-point data analysis by SoftMax Pro software (Molecular Devices, Sunnyvale, CA). The detection limit for TNF-α was 15.6 pg/ml. All measurements were performed in duplicate, and mean values of the two measurements were used for statistical analysis.

### Detection of lipid accumulation in macrophages

#### Accumulation of lipid droplets in macrophages and foam cell formation

For detecting lipid accumulation in macrophages, lipophilic staining by Oil Red O and by BODIPY 493/503 were performed [26], [27]. Macrophages were incubated in growth medium for incubation overnight on chamber slides (Nalge Nunc International). Cells were then treated with ssRNA (2 µg/ml) for 24 h. For Oil Red O staining, neutral lipids were stained using 0.5% Oil-Red-O (Sigma) in isopropanol for 60 min. The Oil- Red-O-stained lipids were morphologically evaluated by microscopy. For BODIPY 493/503 fluorescence staining of cholesterol ester, after incubation for indicated time, the cells were washed twice in ice-cold PBS, followed by paraformaldehyde fixation (2% in PBS) for 1 h at room temperature. The cells were stained with BODIPY 493/503 working solution (10 µg/ml in PBS) for 2 h at room temperature. The cells were rinsed twice with PBS, counterstained with DAPI and mounted on the slides. Micrographs were recorded using an SM 510 META inverted confocal system (Carl Zeiss Microimaging, Inc.) using a 30-mW argon ion laser (excitation wavelength, 488 nm) and emission wavelengths from 505 to 635 nm. To measure lipid accumulation in individual cells, total BODIPY staining intensity per cell was measured from confocal micrographs using SigmaScan Pro 4.0 image analysis software (SPSS Inc). A second method to measure lipid accumulation involved the fluorescence intensity ratio (BODIPY/DAPI) in microplate assays using a Gemini EM spectrofluorometer (Molecular Devices, Sunnyvale, CA). The ratio of BODIPY fluorescence intensity (excitation 490 nm, emission 520 nm, cutoff 515 nm) to DAPI fluorescence intensity (excitation 355 nm, emission 460 nm, cutoff 455 nm) is a readout that measures lipid (BODIPY staining) and normalizes the signal to the number of cells (DAPI nuclear stain).

### Statistical Analysis

Group comparisons were performed using one-way ANOVA followed by *post hoc* analysis using the Dunnett multiple comparisons test. Calculations were performed with InStat3 (GraphPad Software, San Diego, CA) software package. Results are given as mean ± SEM. Statistical significance was accepted for p<0.05.

## Results

### HIV infection is sufficient to induce foam cell formation in monocyte-derived macrophages

**Figure 1:**
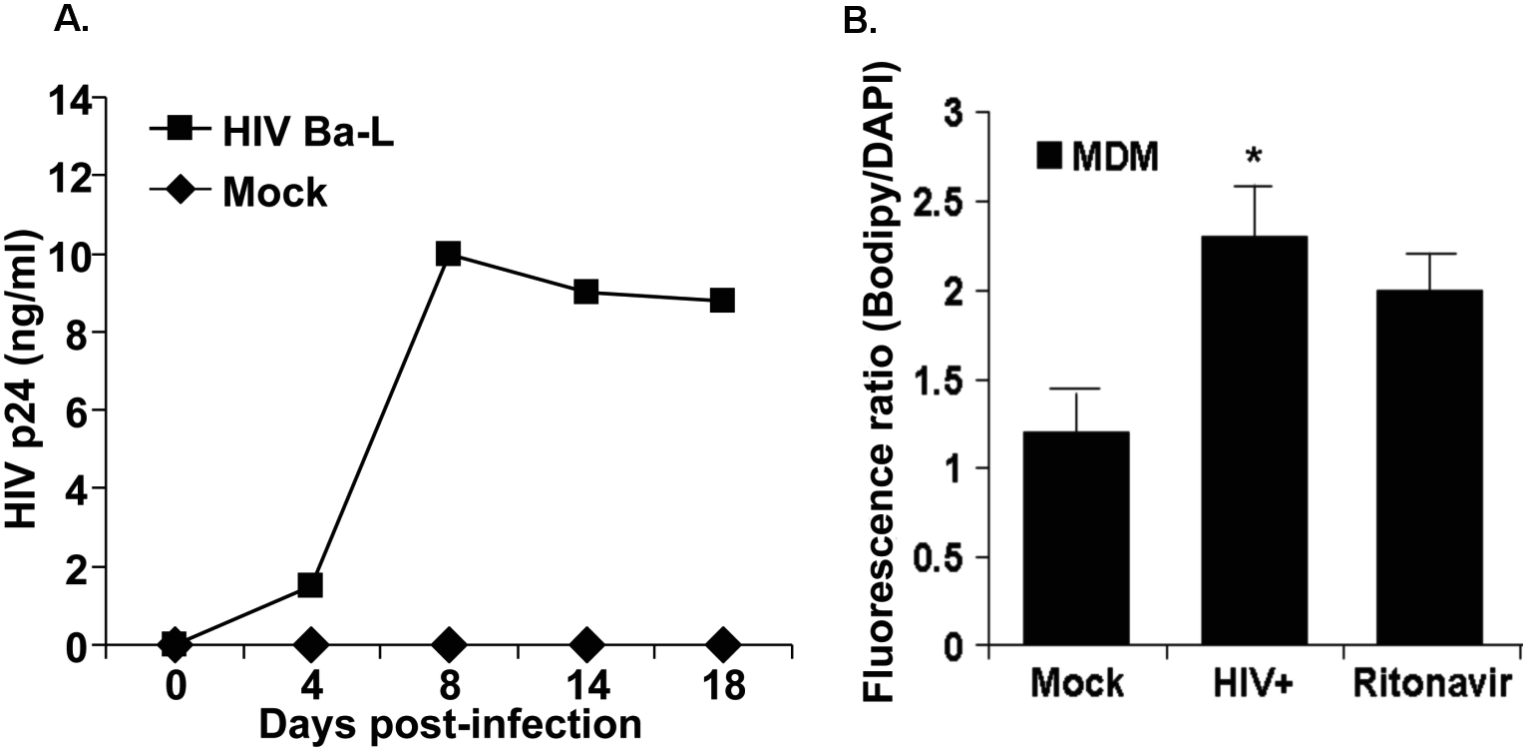
HIV replication and foam cell formation in MDM. **A.** MDMs were exposed to HIV particles, Ba-L strain (10 ng/0.1 Gag p42/10^6^ cells for 3 h and washed. p24 levels were assayed by ELISA. **B.** MDMs were infected with HIV for 4 days and foam cell formation was analyzed by BODIPY staining. Data shown is a representative experiment with similar results from four different healthy uninfected subjects. *p<0.01 compared to mock infected control.

Atherosclerosis incidence is consistently higher among HIV+ patients with or without cART treatment than that in the HIV-negative population [28]. However, emerging data support the hypothesis that HIV infection by itself promotes foam cell formation [29], [30], [7]. To test this hypothesis, we assessed the ability of HIV-1 to stimulate foam cell formation in monocyte-derived macrophages. To determine whether HIV infection *per se* causes foam cell formation, MDM were incubated with HIV-1 particles Ba-L strain (10 ng/0.1 ml Gag p24/10^6^ cells), and cells were washed three hours after incubation. Viral infection was then assessed based on p24 measurements in the supernatant. Viral replication starts at 4 days and peaks at 8 days post infection and plateaus thereafter (Fig. 1A). Importantly, foam cell formation was induced in HIV-infected cells as well as cells treated with a protease inhibitor (PI) ritonavir measured by BODIPY staining (Fig. 1B). These results show that HIV infection alone is sufficient to induce foam cell formation in MDM.

### HIV-1 ssRNA induces foam cell formation in monocyte-derived macrophages human macrophages in a dose-dependent manner

**Figure 2:**
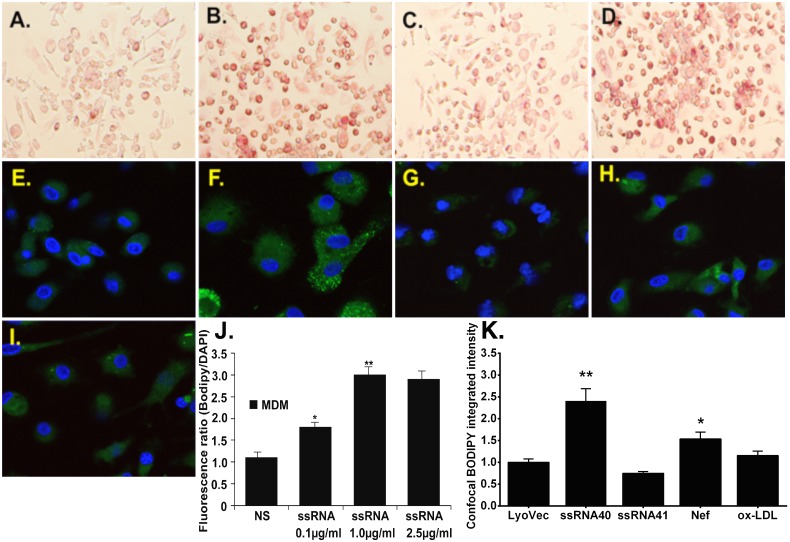
HIV-derived ssRNA induces foam cell formation in a dose-dependent manner. MDMs were stimulated with different stimulants for 24(**A–D**) or BODIPY staining (**E–J**), and the bar graph shows integrated fluorescence intensities for BODIPY in each cell of the confocal micrographs (K). The fluorescence intensity ratio (BODIPY/DAPI) from microplate assays shows the concentration dependence of ssRNA-stimulated lipid accumulation (BODIPY) normalized to the number of cells (DAPI) in **J**. Data shown is a representative experiment with similar results from four different healthy uninfected subjects. *p<0.01, **p<0.01 compared to vehicle control (LyoVec; NS). ssRNA control (ssRNA 41) HIV-ssRNA (ssRNA 40).

Soluble HIV proteins such as Nef, Tat, and Vpr have been detected in the serum of HIV-infected patients [31], [32]. They are probably released into the circulation by infected or apoptotic cells, which may interact with macrophages to drive an inflammatory response. Recently, we have shown that HIV-derived ssRNA40 induced TNFα release in macrophages [33]. We assessed further whether uridine-rich HIV ssRNA is able to induce foam cell formation using two independent methodologies (*ie*. Oil red O staining and BODIPY). HIV-derived ssRNA is sufficient to promote foam cell formation in macrophages as indicated by dose-dependent increases in Oil Red O staining and BODIPY staining (Figs. 2B, F, J). As a negative control, an inactive variant of GU rich ssRNA was synthesized as AU-rich ssRNA. With this change, its addition to macrophages failed to induce foam cell formation. Their appearance was the same as that of unstimulated macrophages (Figs. 2A, C, E, G). These effects were validated by using instead oxidized LDL (ox-LDL), as a positive control, which induces foam formation (Fig. 2D, I). Interestingly, Nef protein (another molecular component of HIV) was also induced foam cell formation in macrophages (Fig. 2H). In addition, confocal BODIPY integrated intensity quantification is shown (Fig. 2K). Taken together, GU-rich ssRNA from HIV is sufficient to induce foam cell formation in macrophages and the effect is specific because when uridine was replaced with adenine this response is lost. These results suggest that increases in foam cell formation by HIV ssRNA could be the result of HIV-induced inflammation.

### Blocking HIV ssRNA-mediated TNFα release inhibits foam cell formation in MDMs

**Figure 3:**
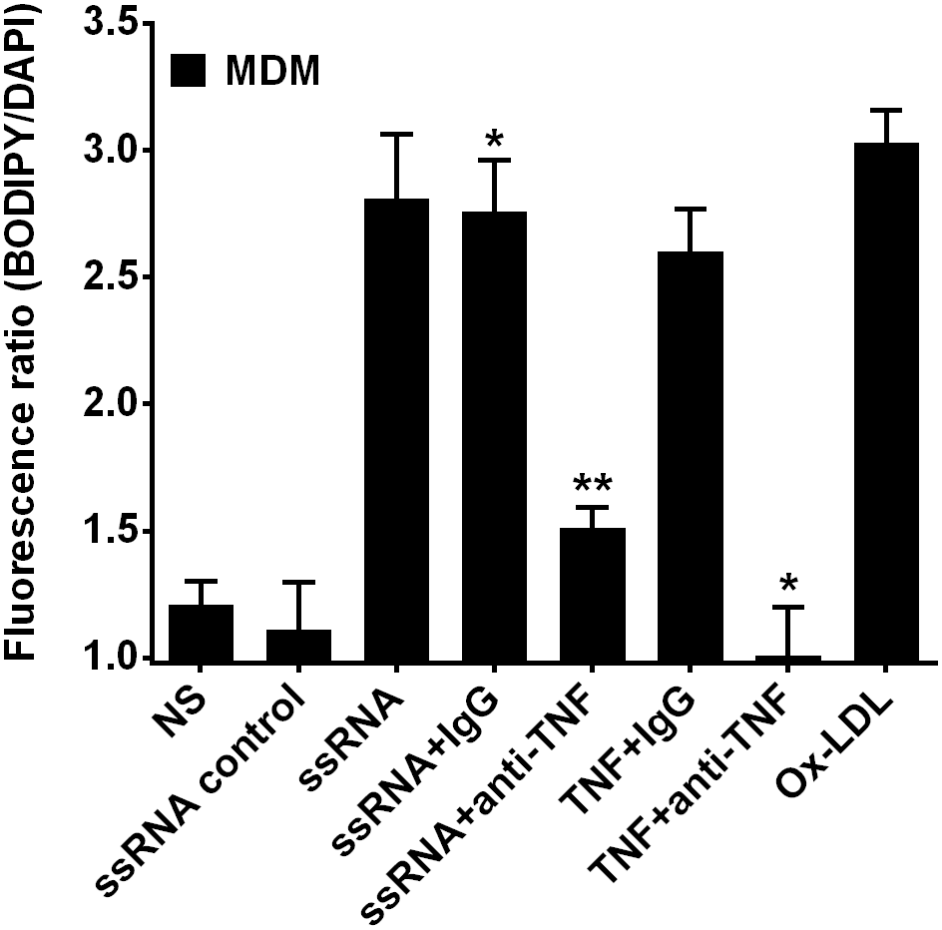
Induction of foam cell formation by HIV-derived ssRNA is dependent on TNFα. MDMs were pre-treated with anti-TNFα for 1 h followed by ssRNA incubation for 24 h and stained with either Oil Red O staining (**A**) or BODIPY staining (**B**). Data shown is a representative experiment with similar results from four different healthy uninfected subjects. *p<0.05 compared to ssRNA control, TNF + IgG, **p<0.01 compared to ssRNA + IgG.

A critical early step in atherosclerosis is the migration of monocytes into the developing atherosclerotic plaques and their development into inflammatory lipid-laden foam cells [29]. We recently demonstrated that signaling by HIV ssRNA induces TNFα release in macrophages [33]. To demonstrate whether inflammatory factors including TNFα mediate foam cell formation, induction of foam cell formation in MDMs was investigated in the absence or presence of neutralizing anti-TNFα antibody. Pre-treatment of human macrophages with anti-TNFα antibody significantly reduced HIV ssRNA40 mediated foam cell formation by 71% (Fig. 3). Activation of TNFα receptor induces robust foam cell formation compared to unstimulated cells (Fig. 3), which was inhibited in the presence of anti-TNFα antibody (Fig. 3). As a positive control, Ox-LDL induced a robust foam cell formation (Fig. 3). These data suggest that inflammatory factors such as TNFα may also act on macrophages to cause them to become foam cells and potentiate early atherogenic processes.

### HIV-1 ssRNA40 induction of macrophage foam cell formation is dependent on endosomal acidification and endocytosis

**Figure 4:**
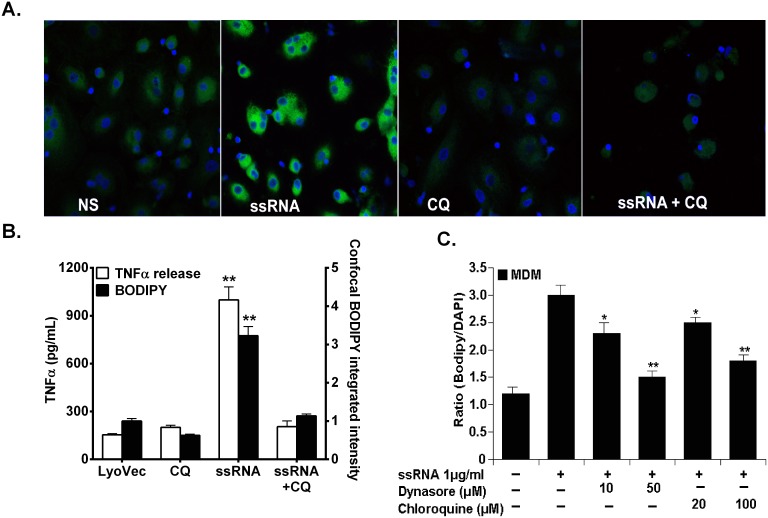
Dependence of HIV-derived ssRNA-induced foam cell formation and TNFα in macrophages on endocytosis and endosomal acidification. MDMs were pretreated with 100 µM chloroquine or 50 µM dynasore for 1 h followed by incubation with 1 µg/ml HIV-ssRNA for 24 h and stained with BODIPY and DAPI (A, C) and cell free supernatant analyzed for TNFα by ELISA and integrated fluorescence intensities for BODIPY in each cell of the confocal micrographs (B). Data shown is a representative experiment with similar results from four different healthy uninfected subjects. *p<0.05, **p<0.01 compared to vehicle control (LyoVec) or HIV-ssRNA alone. NS, non-stimulated vehicle control (LyoVec); CQ, chloroquine; DY, dynasore.

We recently demonstrated that HIV-1 ssRNA signaling induces TNFα release by macrophages that are dependent on TLR8 expression [33]. Next, we determined whether endosomal acidification is required for HIV ssRNA-mediated foam cell formation in MDMs. It has been shown that intracellular TLR signaling is inhibited by chloroquine through inhibition of lysosomal acidification [34]. Pretreatment of MDMs with chloroquine markedly inhibited HIV-ssRNA-mediated foam cell formation (Fig. 4A) and in a dose-dependent manner (Fig. 4C). Interestingly, chloroquine inhibited TNFα release by MDMs down to unstimulated levels (Fig. 4B), suggesting that induction of TNFα by ssRNA40 plays a role in foam cell formation. Next, we investigated the role of endocytosis in HIV-1 ssRNA40 induction of foam cell formation in macrophages, in the presence or absence of an inhibitor of the guanosine triphosphatase (GTPase) dynamin (dynasore). TLR8 is expressed in the luminal aspect of the endosomal membranes; therefore HIV-1 ssRNA40 must undergo endocytosis in order to engage TLR8. Pre-treatment of human macrophages with dynasore significantly reduced foam cell formation induced by HIV ssRNA40 in MDMs in a dose-dependent manner. Reduction of foam cell formation at 10 µM was ∼20% and at 50 µM ∼50% (Fig. 4C), suggesting that dynamin-mediated uptake of HIV ssRNA in MDMs contributes to foam cell formation. Taken together, these data demonstrate in MDMs that HIV-1 ssRNA induces foam cell formation through endocytosis, endosomal acidification and TLR8 signaling.

### HIV-1 ssRNA40 binds TLR8 protein measured by fluorescence resonance energy transfer (FRET) assay

We recently reported that ssRNA40-stimulated TNFα release is dependent upon its recognition and subsequent binding based on the negative results obtained using siRNA-mediated knockdown of TLR8 [33]. However, RNAi knockdown provides indirect evidence of TLR8 involvement. We therefore sought direct evidence of ssRNA40 binding to TLR8 using a fluorescence resonance energy transfer (FRET) assay. The anti-TLR8 antibody clone 44C143 (Imgenex, San Diego, CA) was raised against a KLH-conjugated synthetic peptide from human TLR8 (residues 750–850), which includes an accessible α-helix (residues 781–789) as possible epitope. Based on the published co-crystal structure of CL097 (atom N3) bound to human TLR8 [35], solvent-exposed residues (781–782, 785–786, 788–789) of the α-helix are 65±3 Å away from the bound ligand CL097 (atom N3), which is comparable to the Förster energy transfer radius (*R*_o_ = 64 Å) for the FRET pair Alexa 488 and Alexa 546, [36]. A FRET pair at that distance would have an expected FRET efficiency of 0.47±0.07, which is consistent with the 0.50 FRET efficiency reported for a flow cytometry FRET assay using oligonucleotide- and fluorophore-conjugated antibodies [37]. Since a FRET assay was theoretically feasible, we performed flow cytometry-FRET to detect ssRNA40 binding to TLR8 in a recombinant cell system. Accordingly, HEK-293 cells that had been stably transfected to express human TLR8 were treated with ssRNA40 Alexa 546 conjugate. Cells were fixed, permeabilized and intracellular antigen TLR8 detected using Alexa 488 conjugates of anti-TLR8 antibody or isotype control.

**Figure 5:**
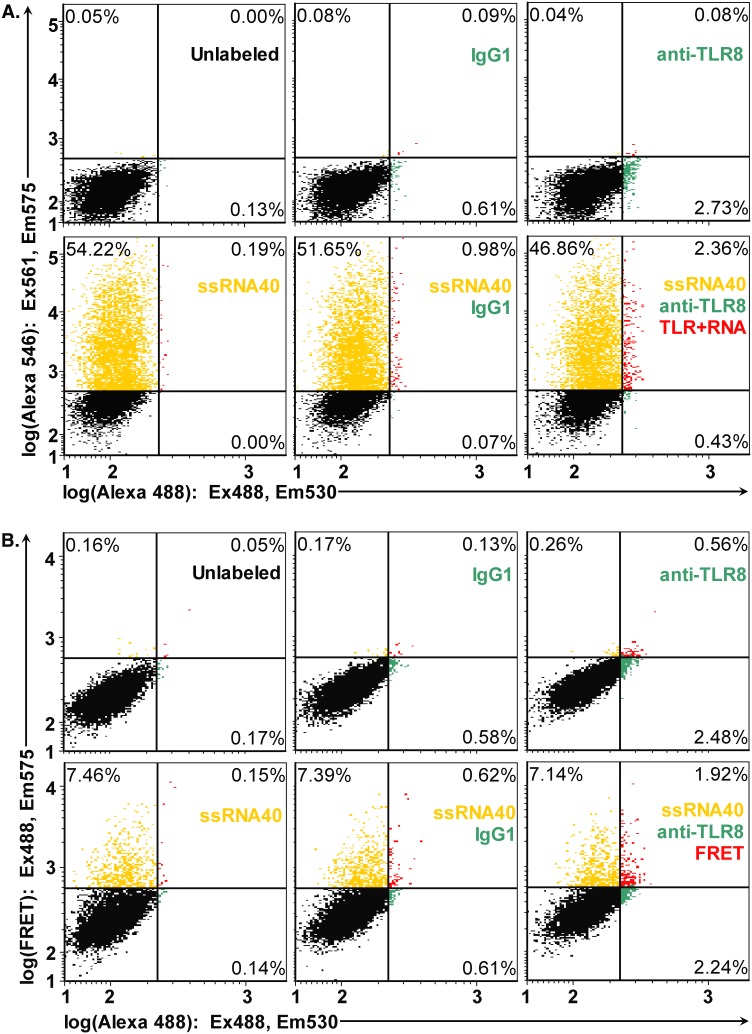
HEK-TLR8 cells take up ssRNA40 (flow cytometry with dual laser excitation). (**A**) HEK cells expressing TLR8 were treated with ssRNA40-Alexa 546 followed by fixation, permeablization and staining for TLR8 using anti-TLR8 Alexa 488 conjugate or isotype control IgG1/κ Alexa 488. TLR8 detection is shown along the horizontal axis, and ssRNA40 Alexa 546 detection is shown along the vertical axis. Direct excitation (488 and 561 nm lasers) of both fluorophores (Alexa 488 and Alexa 546) allowed quantitation of cells staining positive for TLR8 (anti-TLR8-Alexa488) and cells that had taken up ssRNA40-Alexa 546. Single-stranded RNA40 binds TLR8 (flow cytometry FRET assay). (**B**) HEK cells expressing TLR8 were unlabeled or labeled with isotype control antibody (IgG1), anti-TLR8 Alexa 488 conjugate (anti-TLR8) and ssRNA40-Alexa 546 (ssRNA40). Upon excitation at 488 nm, horizontal axes depict Alexa 488 emission (530 nm), and vertical axes show FRET emission (575 nm). Detection of TLR8 is shown in green. Detection of ssRNA binding by TLR8 was measured using emission by the FRET acceptor, Alexa 546 (red). Alexa 546 fluorescence was also observed (D–F, gold). A representative experiment is shown from three independent experiments with similar results.

To evaluate the proportions of labeled cells, we used dual excitation (488 nm and 561-nm lasers). Direct excitation of both fluorophores in conventional flow cytometry demonstrates that HEK-TLR8 had specific labeling with 2.11% of cell population positive for TLR8 (Fig. 5A, anti-TLR8 vs. IgG1 (green)) and 54.2% of cells had taken up the ssRNA40 Alexa 546 conjugate (Fig. 5A, ssRNA40 (gold)). Double-positive cells (1.38% of population were labeled for TLR8 (Alexa 488) and uptake of ssRNA40 Alexa 546 (Fig. 5A, ssRNA40+ anti-TLR8 vs. IgG1+ ssRNA40 (red)).

Cells were further analyzed for FRET by flow cytometry (Fig. 5B) with excitation at 488 nm and emission at 530 nm (FRET Donor; Alexa 488) or 575 nm (FRET Acceptor; Alexa 546). Untreated cells show minimal background fluorescence intensity for both TLR8 (Alexa 488 signal; horizontal axis of Fig. 5B) and for ligand (Alexa 546; vertical axis of Fig. 5B). Isotype antibody contributes little background fluorescence intensity (Fig. 5B, IgG1). HEK-TLR8 cells stained with anti-TLR8 antibody Alexa 488 (FRET acceptor) indicate cells positive for the TLR8 receptor (Fig. 5B, anti-TLR8 (green and red)), whereas isotype control antibody contributes minimal background fluorescence intensity (Fig. 5B, IgG1). HEK-TLR8 cells that had taken up ssRNA40 Alexa 546 (FRET acceptor) were excited at 488 nm showing a fraction of cells (7.46%) were fluorescent (Fig. 5B; ssRNA40 (gold)), which was comparable to 7.39% of cells that had taken up ssRNA40 Alexa 546 followed by staining with isotype control antibody (Fig. 5B, ssRNA40+ IgG1 (gold)). Excitation (488 nm) of HEK-TLR8 cells labeled with both FRET donor and FRET acceptor exhibited total emission within the FRET gate in 1.92% of the cell population (Fig. 5B, ssRNA40+ anti-TLR8 FRET (red)), and subtracting the isotype control background 0.62% (Fig. 5B, ssRNA40+ IgG1 (red)) reveals specific FRET emission in 1.3% of the total cell population. Thus, ∼94% of TLR8-positive cells that had taken up ssRNA40 demonstrated detectable FRET signals indicative of ssRNA40 binding to TLR8 receptor in the flow cytometry FRET assay.

### Foam cell formation in human macrophages is dependent on TLR8 activation by HIV-1 ssRNA40

**Figure 6:**
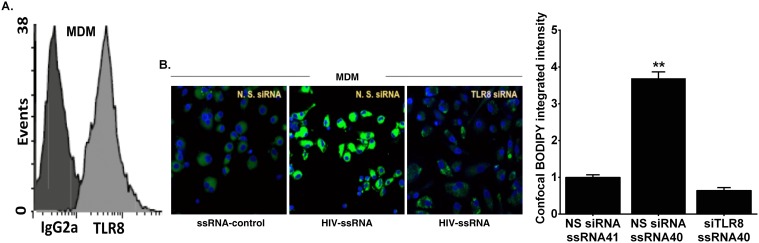
Dependence of foam cell formation in MDMs on TLR8 activation by HIV ssRNA. (**A**) Intracellular expression of TLR8 in MDMs. MDMs which were incubated with PE-conjugated anti-TLR8 or isotype antibody control. Intracellular expression was determined by flow cytometry. Representative profiles were similar in four independent experiments (n = 4 subjects). (**B**) Functional silencing of human TLR8 leads to diminution of foam cell formation in MDMs. BODIPY staining of MDMs after pretreatment with TLR8 siRNA or nonsilencing control. Cells were challenged with HIV ssRNA or ssRNA control for 24 h. Bar graph shows integrated fluorescence intensities of BODIPY per cell of the confocal micrographs. Results are representative of four independent experiments with similar results. **p<0.01 compared to NS siRNA + ssRNA41 control.

As HIV-1 ssRNA derived from Long Terminal Repeats enriched in uridine activate TLR7 in murine dendritic cells and TLR8 in human macrophages, we sought to determine whether TLR8 is expressed in MDMs [38], and we confirmed by flow cytometry analysis that TLR8 is expressed intracellularly in MDMs (Fig. 6A). Accordingly, we next asked whether TLR8 engages HIV-1 ssRNA40 to induce foam cell formation in MDMs. Foam cell formation induced by HIV-1 ssRNA was markedly reduced in TLR8-silenced MDM compared to that in the non-silencing control (Fig. 6B, middle and right panel). Furthermore, an inactive ssRNA41 control failed to induce foam cell formation in non-silenced cells (Fig. 6B, left panel). Taken together, stimulation of foam cell formation by HIV-1 ssRNA40 in MDMs is dependent on TLR8 activation.

### Foam cell formation mediated by HIV-1 derived ssRNA40 in macrophages is dependent on induced MyD88 signaling

**Figure 7:**
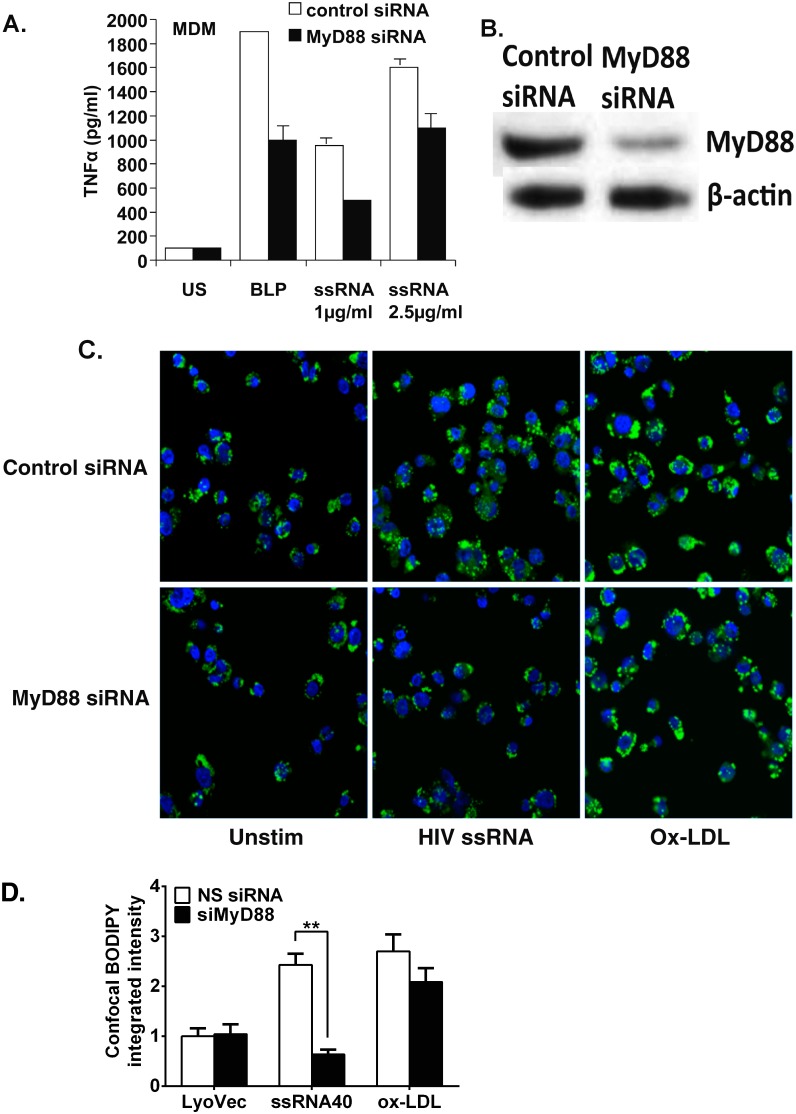
Induction of foam cell formation in MDMs by HIV ssRNA is dependent on MyD88. MDMs were pretreated with MyD88 siRNA and nonsilencing control. Cells were challenged with HIV ssRNA or ssRNA control and incubated for 24α by ELISA (**A**) and BODIPY staining (**C**). Western blot analysis of MyD88 after gene silencing with the use of MyD88 siRNA and nonsilencing siRNA control. β-actin was used to monitor loading after stripping the membrane (**B**). Bar graph shows integrated fluorescence intensities of BODIPY per cell of the confocal micrographs (**D**). A representative blot shows results from one experiment with similar results obtained in four independent experiments.

MyD88 is an adaptor protein that is recruited to mediate TLR8 signaling events leading to the release of cytokines such as TNFα [33]. To determine the involvement of MyD88 in TLR8-linked signaling in MDM cells, foam cell formation and TNFα release were measured in the presence or absence of MyD88-targeted gene silencing. Following exposure to non-silencing siRNA, HIV ssRNA stimulated TNFα release in a dose-dependent manner (Fig. 7A). Similarly, cells transfected with MyD88 siRNA also elicited such a response, but TNFα release was reduced compared to that in non-silenced cells (Fig. 7A). Similarly, TNFα release induced by TLR2 agonist, BLP (10 µg/ml) was diminished in the MyD88-silenced cells. Silencing of *MyD88* gene resulted in ∼65% reduction of MyD88 protein (Fig. 7B). Furthermore, foam cell formation was markedly reduced in MyD88 silenced cells (Fig. 7C, D). To rule out off target effects, foam cell formation is similar in non-silencing control compared to MyD88 silenced cells exposed to Ox-LDL (Fig. 7C). Taken together, in MDMs TLR8 activation by HIV-1 ssRNA stimulates TNFα release and foam cell formation through a MyD88-dependent signaling pathway.

### HIV-derived miRNA induces foam cell formation in a dose-dependent manner and is inhibited by a specific antagomir

**Figure 8:**
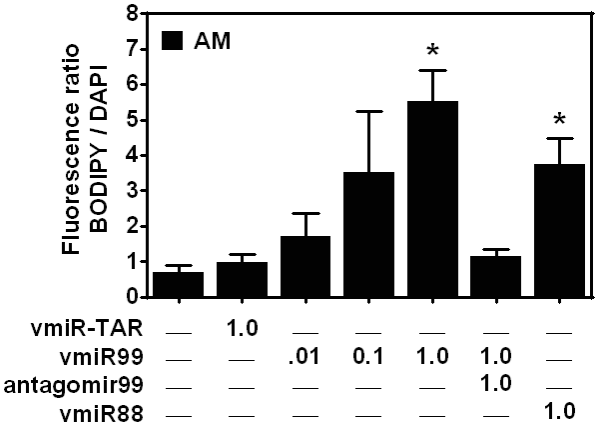
Foam cell formation is induced by HIV vmiR99 in alveolar macrophages in a dose-dependent manner and is inhibited by antagomir99. Alveolar macrophages were plated and pretreated with different doses of antagomir99 followed different doses of HIV vmiR99 and incubated for 24/503 and DAPI. Results are representative of four independent experiments with similar results.

Having established the effects of HIV ssRNA stimulation on foam cell formation, we tested the effect of HIV-derived miRNA on macrophage formation. MicroRNAs are a class of non-coding RNAs consisting of processed products approximately 22 nucleotides in length with canonical function to regulate target gene expression [39], [40]. However, recent studies suggest that miRNAs can even serve as physiological ligands of Toll-like receptors, a function that is independent of their conventional role in post-transcriptional gene regulation [41], [42]. We examined published Deep Sequencing data obtained from HIV-infected cells revealing peaks of short RNA reads throughout the HIV genome [43], and one of these peaks overlaps the ssRNA40 sequence. These GU-rich tract is found in the HIV LTR (R and U5 regions of HIV-1 BaL strain) that encompasses a hot-spot of short RNA indicative of possible mature microRNAs. Using UNAfold RNA-folding software, characteristic shRNA structure was obtained including vmiR-TAR [43], and in addition we identified two novel candidate shRNAs, which we denote vmiR88 and vmiR99. These candidate miRNAs were synthesized and tested for biological activity such as foam cell formation in macrophages, and sequences are shown in the methods section. Incubation of human AM with vmiR-TAR (1 µg/ml) did not induce foam cell formation compared to control (Fig. 8). In contrast, incubation with novel vmiR99 resulted in a dose-dependent induction of foam cell formation. Foam cell formation began at 0.01 µg/ml, and reached a maximum level at 1 µg/ml (Fig. 8). Interestingly, this response was inhibited by antagomir99, which has a completely complementary sequence of its target in a dose-dependent manner. Similarly, vmiR88 also induced a robust foam cell formation (Fig. 8). Taken together, in macrophages HIV-derived miRNA can induce foam cell formation in a dose-dependent manner and can be inhibited by a specific antagomir. These results demonstrate that HIV-derived miRNAs can induce foam cell formation.

## Discussion

In this study, we show in macrophages that HIV infection *per se* is sufficient to induce foam cell formation through TLR8 activation leading to increases in TNFα release. Foam cell formation mediated by HIV ssRNA appears to be driven by inflammatory factors such as TNFα since blocking TNFα down-regulates foam cell formation. Foam cell induction by ssRNA is dependent on dynamin-mediated uptake as well as endosomal acidification. Furthermore, induction of TNFα release by HIV ssRNA is mediated by binding of ssRNA to TLR8, and gene silencing of TLR8 as well as downstream signaling molecules such as MyD88 reduced foam cell formation. These results suggest that inflammatory factors including TNFα induced by HIV-derived ssRNA may activate monocytes to potentiate early atherogenic process in HIV+ individuals.

Chronic immune activation and inflammation have been associated with an increased risk of non-AIDS co-morbidities such as atherosclerosis even among patients on cART. The factors leading to this phenomenon are complex, which includes persistent viral replication coupled with cell death as well as microbial translocation from the gastrointestinal tract. While viral suppression reduces the degree of general immune dysfunction, a chronic inflammatory state persists in many cases [44]. Our finding that foam cell formation in macrophages is inhibited in the presence of an anti-TNFα antibody is an extension of our report describing induction of TNFα release from macrophages in response to HIV ssRNA [33]. These responses agree with the mechanism in which inflammation (i.e. TNFα release) could account for the induction of foam cell formation in macrophages in response to HIV ssRNA. This response is mediated by TLR8 binding to HIV ssRNA inducing TNFα release via MyD88 because silencing of this gene dramatically reduced foam cell formation. These data suggest that HIV-derived ssRNA induces differentiation of monocytes into foam cells by binding to its cognate receptor TLR8, driving MyD88-mediated TNFα release in macrophages and foam cell formation. This early step is critical in the development of atherosclerosis [29].

This sole dependence on HIV infection to induce atherogenic process is evident, since it was induced by exposure to HIV-derived ssRNA TLR8 agonists irrespective of whether this response occurred in healthy monocyte-derived macrophages or primary alveolar macrophages. The fact that this response occurred in HIV+ patients on cART suggests that a chronic inflammatory state persists that elicits an increase in TNFα release to drive foam cell formation. Interestingly, even though foam cell formation is unrelated to whether or not patients received cART, the subsequent underlying pathophysiological changes leading to CVD development in HIV+ patients may differ from those not affected with HIV. Such difference is possible since loci of the pathological changes leading to CVD development are more diffuse in non HIV+ patients than in those who are HIV+ irrespective of whether or not they received cART. The underlying causes for this difference warrants further investigation.

A variety of different TLR subtypes are functionally expressed in macrophages and are activated by pathogens, including bacteria and viruses to induce inflammatory responses [45]. Endosomally restricted TLRs including TLR8 induce inflammatory cytokine release via MyD88 signaling pathway through interaction with ssRNA derived from HIV that has a rich uridine sequence. In addition, it was recently shown that exogenous addition of host miRNAs can induce activation of TLR1 in NK cells [46], suggesting that miRNAs can be recognized by both surface and endosomal TLRs. TLR8 involvement in mediating TNFα release and foam cell formation since function of this endosomal-delimited receptor was compromised by inhibition of endosomal acidification and dynamin-mediated endocytosis with chloroquine and dynasore, respectively. Furthermore, TLR8 and MyD88 gene silencing suppressed HIV ssRNA induction of foam cell formation. Taken together, HIV ssRNA GU-rich oligonucleotide trigger TLR8 activation to induce through MyD88-dependent signaling pathway activation of TNFα release and ultimately foam cell formation. Blockage of this response in a clinical setting may have therapeutic value in suppressing HIV-1 mediated non-AIDS co-morbidities such as atherosclerosis.

In this study, we showed that HIV ssRNA induced TNFα release and foam cell formation could be ascribed to ssRNA binding with TLR8, which is consistent with a recently published report [47]. Foam cell formation appears to be dependent on soluble factors such as TNFα, as our data suggest that blocking TNFα ligation inhibits foam cell formation. These data suggest that inflammatory factors including TNFα and monocytes activation play a role in early atherogenic processes in HIV+ individuals.

In conclusion, this study demonstrates that HIV derived ssRNA induces foam cell formation that is dependent on TNFα release. HIV ssRNA elicits foam cell formation through ssRNA binding with TLR8 and downstream MyD88 activation. MyD88 activation mediated by TLR8 is dependent on endosomal uptake of HIV ssRNA and intravesicular acidification. A better understanding of HIV ssRNA induction of inflammation and ultimately foam cell formation could be a potential target for suppression of chronic immune activation and inflammation.
